# Fine-mapping host genetic variation underlying outcomes to *Mycobacterium bovis* infection in dairy cows

**DOI:** 10.1186/s12864-017-3836-x

**Published:** 2017-06-24

**Authors:** S. Wilkinson, S.C. Bishop, A.R. Allen, S.H. McBride, R.A. Skuce, M. Bermingham, J.A. Woolliams, E.J. Glass

**Affiliations:** 1 The Roslin Institute and R(D)SVS, University of Edinburgh, Easter Bush EH25 9RG, Edinburgh, UK; 2 Agri-Food and Biosciences Institute, Stormont, Belfast, Northern Ireland BT4 3SD UK; 3 School of Biological Sciences, Queen’s University Belfast, Belfast, Northern Ireland BT9 7BL UK; 4 Current Address: Centre for Genomic and Experimental Medicine, School of Molecular, Genetic and Population Health Sciences, University of Edinburgh, Western General Hospital, Crewe Road, Edinburgh, EH4 2XU UK

## Abstract

**Background:**

Susceptibility to *Mycobacterium bovis* infection in cattle is governed in part by host genetics. However, cattle diagnosed as infected with *M. bovis* display varying signs of pathology. The variation in host response to infection could represent a continuum since time of exposure or distinct outcomes due to differing pathogen handling. The relationships between host genetics and variation in host response and pathological sequelae following *M. bovis* infection were explored by genotyping 1966 Holstein-Friesian dairy cows at 538,231 SNPs with three distinct phenotypes. These were: single intradermal cervical comparative tuberculin (SICCT) test positives with visible lesions (VLs), SICCT-positives with undetected visible lesions (NVLs) and matched controls SICCT-negative on multiple occasions.

**Results:**

Regional heritability mapping identified three loci associated with the NVL phenotype on chromosomes 17, 22 and 23, distinct to the region on chromosome 13 associated with the VL phenotype. The region on chromosome 23 was at genome-wide significance and candidate genes overlapping the mapped window included members of the *bovine leukocyte antigen class IIb* region, a complex known for its role in immunity and disease resistance. Chromosome heritability analysis attributed variance to six and thirteen chromosomes for the VL and NVL phenotypes, respectively, and four of these chromosomes were found to explain a proportion of the phenotypic variation for both the VL and NVL phenotype. By grouping the *M. bovis* outcomes (VLs and NVLs) variance was attributed to nine chromosomes. When contrasting the two *M. bovis* infection outcomes (VLs vs NVLs) nine chromosomes were found to harbour heritable variation. Regardless of the case phenotype under investigation, chromosome heritability did not exceed 8% indicating that the genetic control of bTB resistance consists of variants of small to moderate effect situated across many chromosomes of the bovine genome.

**Conclusions:**

These findings suggest the host genetics of *M. bovis* infection outcomes is governed by distinct and overlapping genetic variants. Thus, variation in the pathology of *M. bovis* infected cattle may be partly genetically determined and indicative of different host responses or pathogen handling. There may be at least three distinct outcomes following *M. bovis* exposure in dairy cattle: resistance to infection, infection resulting in pathology or no detectable pathology.

**Electronic supplementary material:**

The online version of this article (doi:10.1186/s12864-017-3836-x) contains supplementary material, which is available to authorized users.

## Background

Bovine tuberculosis (bTB), caused by *Mycobacterium bovis*, a member of the *Mycobacterium tuberculosis* complex, is a disease that adversely affects cattle in many parts of the world. The zoonotic pathogen impacts the health and welfare of cattle and there is the additional risk of spread of infection to other mammalian species. Prevalence of bTB has increased in the United Kingdom since the mid-1980s, particularly in the higher risk areas of Wales and South-West of England [1]. To tackle bTB the government currently implements active surveillance and bTB eradication measures including routine diagnostic testing of herds using the tuberculin skin test, culling of test reactor animals, movement restrictions on infected herds and abattoir surveillance. However, despite the eradication programme recent trends indicate that bTB continues to persist in the UK [1].

Resistance to *M. bovis* infection in cattle is complex, with quantitative genetic studies showing that the trait is influenced, in part, by host genetic variation. Moderate heritable variation for bTB resistance has been found for both dairy [2, 3] and beef populations [4], indicating that breeding for increased bTB resistance in cattle is a viable strategy to reduce the prevalence and spread of the disease in the national herds. A recent study on the prediction of disease susceptibility in dairy cows using genetic markers demonstrated that genomic selection for bTB resistance is feasible and could therefore be complementary to current control measures [5]. In addition, dissection of the genomic architecture of the trait has revealed many bTB resistance loci mapped to several chromosomes for different cattle populations [6–9].

After establishment of infection a range of outcomes can manifest in the host. It is recognised that human tuberculosis (TB) infection, caused by the highly-related mycobacterium, *M. tuberculosis*, is manifest in at least two clinical phenotypes, latent and active TB [10] with most infected humans classified as latent with no clinical symptoms, and around 10% developing symptoms, most commonly as a pulmonary disease. Furthermore, Young et al. [11] proposed that the host TB spectrum could be extended to include an asymptomatic subclinical state and a wider range of host responses including clearance of infection through innate or acquired immune mechanisms [11]. Many factors play a role in determining how exposed individuals respond to infection and whether they ultimately succumb to clinical disease. Given the complexity of the host-pathogen interactions that occur during infection with *M. tuberculosis*, it is not surprising that host genetics also plays a role in influencing the outcome of infection [12]. However, reflecting the difficulties in testing directly for infection with or exposure to *M. tuberculosis*, the majority of human genetic studies have focused on susceptibility to pulmonary disease as this phenotype is easier to diagnose [12]. Nonetheless, attention is now turning to the suggestion that potentially distinct genetics may also control other measureable phenotypes within the host TB spectrum including skin test reactivity in humans [12–15].

A spectrum of host phenotype responses could also be considered for bTB [16]. Similar to human TB, there is observed variation in pathology amongst cattle infected with *M. bovis* [17]. In addition, as in humans, the bTB diagnostic tests are limited and show variation in specificity and sensitivity and a lack of correlation between tests (reviewed by Strain et al., [18]). For instance, all animals diagnosed as infected using the single intradermal comparative cervical tuberculin (SICCT) test undergo carcase abattoir examination for signs of disease pathology and typically around 30 to 40% of these are confirmed for *M. bovis* infection in the form of tubercle lesions (VL) [19]. The remaining 60–70% of animals that are skin-test positive but not displaying detectable signs of pathology at carcase abattoir examination (NVL) could be expressing a phenotype that is indicative of a time lag due to early stage infection, a state of latency or other environmental factors such as low dose of pathogen challenge [17]. Additionally, the NVL diagnosis may also represent a divergent host response phenotype under distinct host genetics.

This paper explores these hypotheses by extending an earlier VL case–control genome-wide association study (GWAS) on bTB resistance [6] to quantify genetic variation associated with the NVL phenotype and compare with data on the VL phenotype. The aim of the study is to dissect the genetic architecture of the outcomes of *M. bovis* infection in dairy cattle.

## Results

### Regional heritability (RH) mapping

RH mapping was conducted [20] to identify regions of genetic variance associated with *M. bovis* infection outcomes. A previous bTB case–control study, reported a quantitative trait locus (QTL) on BTA13 associated with the VL phenotype (controls vs VLs) detected by RH mapping [6]. The extra 19 re-genotyped VLs in the current study produced concordant results and therefore these results are not reported further.

**Fig. 1:**
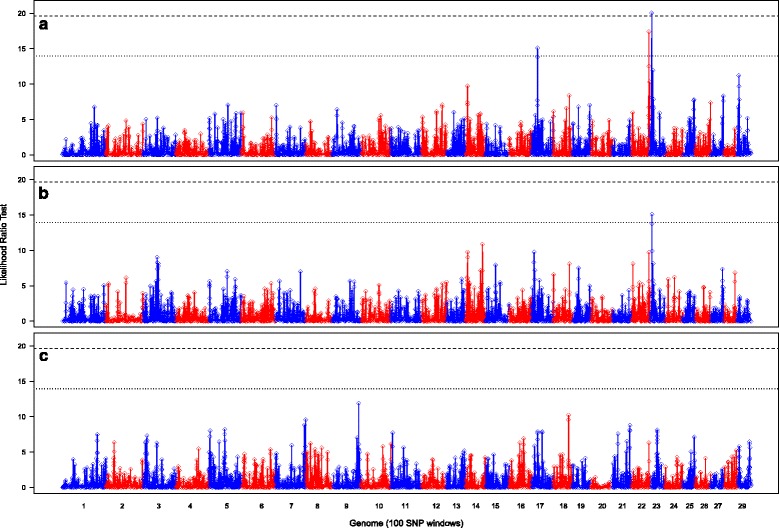
Regional heritability for bTB resistance. Shown is the likelihood ratio test (LRT) across the genome using 100-SNP windows for **a** controls vs NVLs, **b** control vs cases and **c** NVLs vs VLs. Genome-wide (*P* < 0.05) and suggestive significance (one false positive per genome scan) LRT thresholds are shown as a dashed and dotted line, respectively

**Fig. 2:**
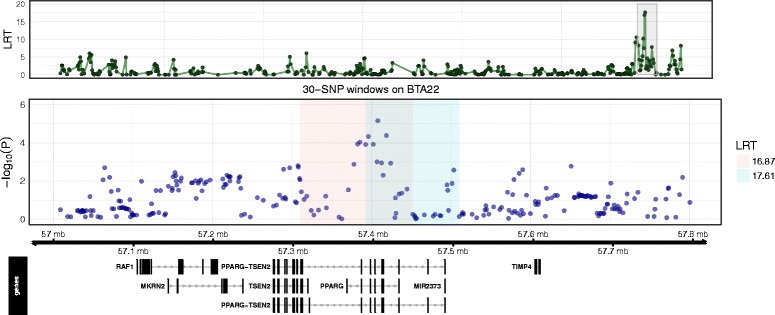
Associations at the region on BTA22 detected in the NVL phenotype (controls vs NVLs). The top panel shows RH mapping results using 30-SNP windows with the LRT plotted with respect to genomic position on BTA22. A *grey* rectangle highlighting 30-SNP windows significant at the suggestive level. The middle panel shows the associations (−log_10_(*P-*value)) from the GWAS for SNPs located within and flanking the RH mapped region of significance. The *shaded colour* boxes in this panel indicate the genomic position of the RH mapped region of significance with corresponding LRT values displayed in the legend box to the right. The bottom panel shows the protein coding regions within and flanking the RH mapped region of significance. These were obtained using the bioconductor annotation R package [53] with the full genome sequence for *Bos taurus* provided by UCSC [54]

**Fig. 3:**
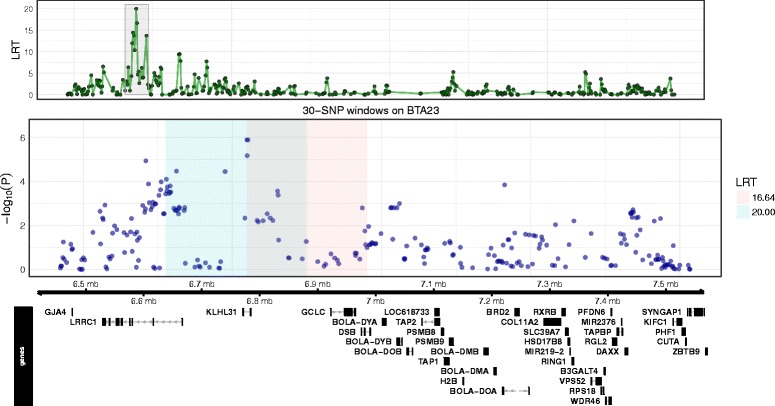
Associations at the region on BTA23 detected in the NVL phenotype (controls vs NVLs). The top panel shows RH mapping results using 30-SNP windows with the LRT plotted with respect to genomic position on BTA23. A *grey* rectangle highlighting 30-SNP windows significant at the suggestive level. The middle panel shows the associations (−log_10_(*P-*value)) from the GWAS for SNPs located within and flanking the RH mapped region of significance. The *shaded colour* boxes in this panel indicate the genomic position of the RH mapped region of significance with corresponding LRT values displayed in the legend box to the right. The bottom panel shows the protein coding regions within and flanking the RH mapped region of significance. These were obtained using the bioconductor annotation R package [53] with the full genome sequence for *Bos taurus* provided by UCSC [54]

Regions of genetic variance associated with NVL phenotype (controls vs NVLs), cases (controls vs VLs and NVLs) and NVLs vs VLs detected by RH mapping using 100-SNP windows are presented in Fig. 1 and Table 1. The region on BTA13 previously identified for the VL phenotype by Bermingham et al., [6] was not detected for the NVL phenotype using RH mapping. Single SNP-based GWAS identified two SNPs at suggestive significance on BTA13 associated with the NVL phenotype, but they were approximately 7 Mb and 13 Mb distant from the QTL reported by Bermingham et al., [6] (Additional files 1 and 2). Instead, RH mapping identified three QTLs distinct to NVLs, two regions at suggestive significance and one region at genome-wide significance (Fig. 1a). The first region at suggestive significance was on BTA17 spanning 19.34–19.65 Mb. Within this region was one uncharacterised gene, a pseudogene, and nearby was the solute carrier *SLC7A11*, which fell within an adjacent overlapping window that was just below suggestive significance (19.51–19.83 Mb, LRT = 13.86, h^2^_r_ = 0.044). Fine RH mapping of the associated region on BTA17 failed to resolve this QTL as no regions were detected at genome-wide or suggestive significance. The second region at suggestive significance was located at 57.16–57.54 Mb on BTA22 comprising a moderately gene-rich region with six genes found. Fine RH mapping of the associated region improved the resolution of the QTL, revealing two overlapping 30-SNP windows at suggestive significance located at 57.31–57.51 Mb (Fig. 2). Within the two 30-SNP windows were the genes *TSEN2* (57.27–57.32 Mb), *PPARG* (57.36–57.43 Mb) and *MIR2373* (57.48–57.49 Mb) and SNPs were found to be just below the suggestive significance level for the single SNP-based GWAS (Fig. 2). The third region, which reached genome-wide significance level, was on BTA23 extending from 6.60–7.06 Mb. This region was relatively gene rich and included members of the *bovine leukocyte antigen* (*BoLA*) *class IIb* (6.97–7.53 Mb) also falling under the significant window. Fine RH mapping of the associated region revealed two overlapping 30-SNP windows at suggestive significance (Fig. 3). Within these windows there were also two SNPs at suggestive significance at 6.774 Mb and 6.777 Mb detected by the single SNP-based GWAS (Fig. 3; Additional files 1 and 2). Within the region were the genes *KLHL31* (6.77–6.78 Mb), *GCLC* (6.92–6.96 Mb) and *DSB* (6.97–6.99 Mb).

**Table 1:** Genomic regions associated with bTB resistance

	chr	Position (bp)	LRT	*h* ^2^_r_	Candidate Genes
NVLs	*100-SNP windows*				
	17	19,342,437–19,650,334	15.10	0.053	LOC783390 matrin 3 pseudogene
	22	57,159,725–57,547,638	17.42	0.039	RAF1, MKRN2, MKRN2OS, TSEN2, PPARG, MIR2373
	23	6,603,103–7,066,824	20.05	0.035	LRRC1, LOC104975626, KLHL31, GCLC, DSB, BOLA-DYA, BOLA-DYB, BOLA-DOB, LOC100140517
	*30-SNP windows*				
	22	57,311,814–57,451,910	16.87	0.026	TSEN2, PPARG
	22	57,396,014–57,510,400	17.61	0.025	PPARG, MIR2373
	23	6,669,083–6,873,884	20.00	0.041	LOC104975626, KLHL31
	23	6,774,875–6,975,825	16.64	0.046	KLHL31, GCLC, DSB
Cases	100-SNP windows				
	23	6,411,854–6,774,223	15.12	0.033	MLIP, LRRC1, LOC104975626, KLHL31

For the associated trait, the table shows chromosome, start and end position of genomic window in base pairs, LRT, heritability of the region and candidate genes residing within the window of significance. Genomic regions at both the genome-wide and suggestive level identified by regional heritability mapping using 100- and 30-SNP window size are presented

RH mapping of all cases (VLs and NVLs) revealed one window at suggestive significance, located on BTA23 at 6.41–6.77 Mb (Fig. 1b). This window overlapped those detected on BTA23 for the NVL phenotype and downstream from this window is the *BoLA class IIb* region. Fine RH mapping of the whole genome using 50- and 30-SNP windows resulted in no windows at suggestive or genome-wide significance.

Considering the comparison between the NVL and VL phenotypes (NVLs vs VLs), RH mapping using a 100-SNP window failed to detect regions at the genome-wide or suggestive significance levels (Fig. 1c). Regions just below the suggestive significance level were located on BTA7 (111.52–111.78 Mb, LRT = 9.57), BTA9 (98.14–98.53 Mb, LRT = 11.88) and BTA18 (49.40–49.83 Mb, LRT = 10.21). Fine mapping of these chromosomes detected a 30-SNP window at suggestive significance on BTA9 (98.34–98.49 Mb, LRT = 18.02, h^2^_r_ = 0.030). There were three candidate genes located within the 100-SNP window, *MAP3K4* (98.12–98.23 Mb), *AGPAT4* (98.23–98.37 Mb) and *PARK2* (98.42–98.45 Mb) with the third gene residing within the 30-SNP window. No significant SNPs from the GWAS analysis were found within the top RH mapped windows (Additional files 1 and 2).

### Chromosome heritability

The variance attributable to each chromosome was estimated using a single chromosome decomposition that included a genome-wide polygenic effect of the remaining SNPs (i.e. the model fitted a GRM for the target chromosome along with a whole-genome GRM constructed for all other chromosomes excluding the target chromosome).

**Fig. 4:**
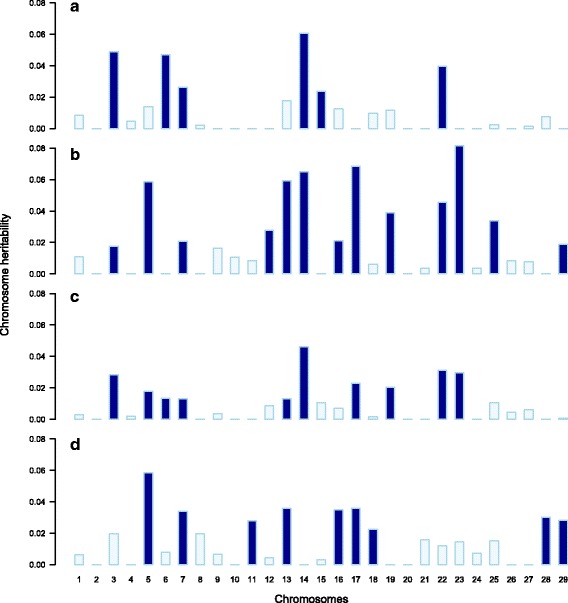
Proportion of phenotypic variance explained by each chromosome for bTB resistance. Shown are the chromosome heritability estimates for **a** controls vs VLs, **b** controls vs NVLs, **c** controls vs cases and **d** NVLs vs VLs. *Dark blue* bars represent chromosome heritability estimates greater than their standard errors and lightly *shaded blue* bars represent chromosome heritability estimates less than their standard errors

For the case phenotype VLs (controls vs VLs), six chromosomes explained a proportion of phenotypic variance (Fig. 4a). In contrast, variance was attributed to thirteen chromosomes for the NVL phenotype (Fig. 4b). Four of these chromosomes, BTA3, BTA7, BTA14 and BTA22, were also found to explain a proportion of phenotypic variance for the VLs (controls vs VLs). Confirming RH mapping, the highest chromosomal heritability estimates for the NVLs were chromosomes with detected QTLs, i.e. BTA17, BTA22 and BTA23.

When the two *M. bovis* infection phenotypes were grouped together (controls vs all cases), variance was attributed to ten chromosomes (Fig. 4c). Of these chromosomes, BTA3, BTA7, BTA14 and BTA22 were found to explain a proportion of phenotypic variance when the case phenotype was either the VLs or the NVLs. Of the six other chromosomes, BTA5, BTA13, BTA17, BTA19 and BTA23 explained a proportion of phenotypic variance for the NVLs and BTA6 explained a proportion of phenotypic variance for the VL phenotype.

Considering NVLs vs VLs, variance was attributed to nine chromosomes (Fig. 4d). Five of these chromosomes distinguished the two case phenotypes, in that they explained a proportion of phenotypic variance for the NVLs but not for the VLs (BTA5, BTA13, BTA16, BTA17 and BTA29). Three additional chromosomes also explained a proportion of the phenotypic variance for one of the case phenotypes but the estimates were not significantly different from zero (BTA28 for the VLs and BTA11 and BTA18 for the NVLs). Heritable variation was found for BTA7 when the phenotype considered was either VLs, NVLs or all SICCT positive cases.

There was no evidence for a linear relationship between chromosome length and chromosome heritability (controls vs VLs, *P* = 0.28 and *R* ^2^ = 0.007; controls vs NVLs, *P* = 0.29 and *R* ^2^ = 0.005; controls vs cases, *P* = 0.67 and *R* ^2^ = −0.03; NVLs vs VLs, *P* = 0.89 and *R* ^2^ = −0.036).

### Genome wide association study

Results from the genome-wide association analysis are reported in the supplementary material (Additional files 1, 2, 3, 4 and 5). Briefly, whilst SNPs were significantly associated with the NVLs (controls vs NVLs), cases (controls vs cases) and NVLs vs VLs there were no clear regions of association detected because significant SNPs were not neighbouring or in close proximity to one another.

## Discussion

Regional heritability (RH) mapping and chromosomal heritability revealed a number of genomic regions and chromosomes with heritable variation significantly associated with the *M. bovis* infection outcomes defined in this study. The genetic architecture underlying the two case phenotypes (VLs and NVLs) appears to be partly distinct, although they are highly genetically correlated. Our results suggest there are (at least) two distinct outcomes following *M. bovis* infection in dairy cattle. In addition, the chromosomal variance associated with each *M. bovis* infection outcome and all cases was not apportioned across the entire bovine genome and the proportion of variance explained by each chromosome length was not proportional to its length. This is consistent with a moderately polygenic model, suggesting that bTB resistance is under the control of a large number of regions with variants of small effect size distributed across certain chromosomes. The controls were animals that were SICCT test negative on multiple occasions following herd breakdown and thus they appear able to resist or clear *M. bovis* infection without resorting to a detectable acquired immune response.

### Mapped QTLs associated with bTB resistance

GWAS and RH mapping, respectively, identified a number of SNPs and regions associated with bTB resistance. However, strong and informative QTL effects were not detected by the single SNP-based GWAS as the significant SNPs were singletons (prompting the perception that they may be false positives). RH mapping combines the effects of neighbouring SNPs into a single estimate of heritability, thereby increasing the power of detecting a QTL otherwise tagged by individual SNPs of small effect [20]. As observed in other studies [21, 22], RH mapping identified additional loci undetected by single SNP-based GWAS.

The strongest QTL detected by RH mapping and the only one at genome-wide level for NVLs (vs controls) was found on BTA23. In addition, the GWAS revealed two SNPs at suggestive significance residing within this region. Genes underlying this QTL included glutamate cysteine ligase catalytic subunit (*GCLC*) and members of the *bovine leukocyte antigen* (*BoLA*) *class IIb* region. *GCLC* is a rate-limiting enzyme in the glutathione synthesis pathway and has been found to be differentially expressed in bovine macrophages in response to pathogens, including *Salmonella enterica* [23] and *M. bovis* (unpublished work, pers. comm. K Jensen). The *BoLA class IIb* region is part of the *BoLA* complex, the bovine equivalent of the Major Histocompatibility Complex (MHC), which plays a central role in adaptive immunity. *BoLA* genes encode cell surface molecules that process and present pathogen peptide fragments (antigens) to T cells [24]. The *DY* genes, which are at the start of the *class IIb* region and were found residing in the QTL detected for the NVL phenotype, are unique to ruminants and may have a specialised role in antigen presentation to ruminant dendritic cells [25]. The other *BoLA* classes, including *class IIa*, are separated from *class IIb* by ~17 Mb of sequence on BTA23 due to an historical inversion event [26]. Although no associations with *M. bovis* susceptibility were detected for the other *BoLA* classes in the current or other bTB studies, it is clear that the *BoLA class II* alleles influence recognition of *M. bovis* T cell epitopes [16]. Furthermore, the concentration of polymorphic immune-related loci on BTA23 may account for it possessing the highest chromosome heritability for NVLs, explaining 7.7% of the phenotypic variation of bTB resistance. In addition, the current bovineHD chip has relatively sparse coverage of SNPs across the *BoLA* complex and poor assembly of this genome region may explain the lack of detectable QTL for bTB resistance in other regions on BTA23 in this and previous bTB GWAS studies.

This QTL on BTA23 was also detected for all cases (controls vs cases), albeit at the suggestive level. It was not detected in VLs, nor did the chromosome as a whole explain a proportion of the phenotypic variation for the VL phenotype suggesting this QTL may be associated with an *M. bovis* infection outcome with no detectable pathology. This region was within 3 Mb of another highly significant bTB resistance QTL recently reported by Richardson et al., [8], a study using estimated breeding values (EBVs) for bTB resistance derived from SICCT data in a cattle population distinct from our study. Thus, this region of the genome could be a prime candidate associated with bTB resistance in dairy cattle.

A second strong QTL, only detected in NVLs, was on BTA22, approximately 1 Mb upstream from a QTL reportedly associated with bTB resistance EBVs [7]. Fine mapping of the QTL associated with the NVL phenotype resolved a 70 kb block and of the genes in the window peroxisome proliferator-activated receptor gamma (PPAR-γ) was identified as a likely candidate gene because of evidence of a role in immunity. PPAR-γ is a lipid sensing receptor expressed by macrophages and dendritic cells [27] and up-regulated in the presence of *M. tuberculosis* [28]. It has been shown to detect *M*. *tuberculosis* lipids in infected macrophages leading to inhibition of phagolysosome maturation and enhanced survival of the bacteria [29]. Thus, *PPAR*-γ associated variants may play a role in the variation in bTB outcome following infection in dairy cattle.

Additional putative regions associated with bTB resistance were detected on BTA17 and BTA9, the former associated with the NVL phenotype and the latter found to distinguish the two *M. bovis* infection outcomes (NVLs vs VLs). Both these QTLs were novel to this study, but the evidence of association was relatively low, thus there is the possibility that they were false positives. In addition, the QTL on BTA17 occurred in a relatively gene poor region and whilst the QTL on BTA9 contained a few genes there were no obvious candidates previously associated with disease resistance.

The bTB resistance QTL associated with the VLs (vs controls) on BTA13 reported by Bermingham et al., [6] was not detected for the NVLs, all cases or when contrasting the NVLs with the VLs using the single SNP-based GWAS and window-based RH mapping. Similarly, studies where the phenotype was expressed as bTB resistance EBVs derived from SICCT records (therefore using both VLs and NVLs to obtain the phenotype) also did not detect this QTL [7, 8]. The results suggest that the QTL on BTA13 may only be associated with detectable bTB pathology in dairy cattle.

### The host genetics of infection outcome with *M. bovis*

Taking all of the evidence together it would appear that there are at least three potential outcomes following exposure to *M. bovis* in dairy cattle. There is a group of animals that are resistant to infection and may employ innate immune-related mechanisms to prevent establishment of *M. bovis* and thus are able to eradicate the pathogen without invoking the delayed type hypersensitivity response that is measured by the SICCT test or interferon-γ assay [30]. Similarly, many humans exposed to high risk of infection with the related pathogen, *M. tuberculosis* do not become infected, and a specific region of the human genome has been associated with both SICCT test negativity and a major innate immune pathway associated with infection and progression of human TB [31]. Potentially, the control resistant cattle employ similar innate immune defence mechanisms and further research in this area would be warranted.

The second and third group of susceptible animals are unable to prevent establishment of infection, as evidenced by their positive reaction to the SICCT. However, the outcome following infection is variable in cattle [17, 32] and the evidence presented in this paper would suggest that there are at least two outcomes to *M. bovis* infection: detectable disease (VLs) versus no detectable visible lesions (NVLs), which are under some distinct genetic control. One hypothesis considered is that the NVLs were simply animals tested at early stages of infection with lesions too small to detect under the naked eye. If so, it was anticipated that the underlying genetics of susceptibility would be indistinguishable for the two *M. bovis* infection phenotypes. Instead, different loci were associated with VLs (versus controls) compared to NVLs, and there was additional variance explained by chromosomes for the NVLs not found for the VLs. Second, genetic differences were also detected when the VLs were contrasted with the NVLs. There was moderate genome-wide SNP heritability detected between the VLs vs NVLs (h^2^= 0.29 (± 0.06)) and chromosomes were found to harbour variants that distinguished the two case phenotypes. However, a degree of genetic control underlying bTB resistance appears to be shared between the two *M. bovis* infection outcomes. When grouped together (and contrasted against the controls) variance was attributed to several chromosomes and the genome-wide SNP heritability was moderate (h^2^= 0.22 (± 0.04)).

Since the traits defined in this study are dependent on statutory screening tests performed on the national herd, their properties could affect the categorisation of animals into the three phenotypes considered here. The SICCT test was used to first classify dairy cows as cases (VLs and NVLs) and controls. The test has a high specificity for both the standard and severe interpretations [33, 34], which is sufficiently high to result in a high positive predictive value (PPV). In addition, even with this small subset of false positives for the SICCT test, there is only very small heritability in the liability to test positive when healthy [35]. Therefore, it is highly likely that with a positive reaction to the SICCT test, all of the cases, which constitute both the VL and NVL phenotypes, are likely infected with *M. bovis* and the genetic variation associated with bTB arises from infection. In contrast to specificity, the sensitivity of the SICCT test is moderate with a range of 50–80% for a single test [33, 36–38], suggesting a small proportion of the controls may be false negatives. The design of the current study had strict criteria for selecting controls maximising that they were healthy exposed animals: identified animals were negative on multiple occasions for the SICCT test herd breakdown and were also subjected to retrospective checks to ensure their continuing healthy status. Furthermore, herds undergoing breakdowns are subject to repeated short-interval testing and remain closed until the whole herd is SICCT negative, thus the breakdown process has a high sensitivity, removing infected animals and leaving the uninfected ones in the herd. Despite the imperfect SICCT test properties, genetic variants associated with *M. bovis* susceptibility were detected for the case phenotypes (when contrasted against controls), which supports the criteria used to diagnose the cases and controls in this study.

The second diagnostic test used in this study was carcase abattoir examination for signs of pathology to classify cases (SICCT-positives) as VLs or NVLs. This test has a high specificity because the majority (95–100%) of visibly lesioned SICCT test reactors are also culture positive for *M. bovis* [39], supporting the VL infection status. However, the sensitivity of carcase abattoir examination is moderate at best, at around ~45% [40]. This implies that a proportion of SICCT-positives with lesions may not be confirmed as VLs under examination, but instead are misclassified as NVLs. It is argued that the sensitivity of lesion detection is compromised by prevailing abattoir conditions, such as limited time and visual rather than microscopic inspections [40, 41]. If so, an NVL diagnosis would represent an early stage of infection not yet progressed to the disease presentation observed in the VL phenotype. With the hypothesis that sensitivity of carcase examination is influenced to a large degree by abattoir conditions, little genetic differences would be expected between the NVL and VL phenotype. However, variants unique to each case phenotype and chromosomal variation distinguishing the NVLs and VLs were observed, suggesting there may be at least two host phenotype responses to *M. bovis* infection in dairy cattle. Variation in pathology of SICCT-positives may also be partly genetically determined and indicative of different host responses or pathogen handling. Thus, a proportion of animals with the NVL phenotype may be able to control and contain *M. bovis*, an outcome which may lead to latent infection [32].

Distinct genetic loci may also play a role in the observed spectrum of *M. tuberculosis* infection phenotypes in humans with recent candidate gene case–control studies identifying polymorphisms in *IL10* [42] and *TLR9* [43] associated with latent TB but not active TB. In addition, a human TB GWAS, incorporating a number of infection outcomes, identified SNPs in the *HLA* class II region significantly associated with pulmonary TB and *M. tuberculosis* infection whilst no variants were significantly associated with all confirmed TB cases [14].

### Breeding for bTB resistance in cattle

The existence of a host genetic component to bTB resistance in dairy cattle is well established. Thus, a genetic strategy to reduce bTB prevalence is a credible option, using pedigree-based EBVs and/or a whole genome selection approach (gEBVs). Although the genetic results in the present study suggest the host genetics underlying the two *M. bovis* infection outcomes are governed, in part, by distinct genetic variants, variance was attributed to many chromosomes for all cases (VLs and NVLs), altogether capturing 24% of the phenotypic variation. Thus, when using the SICCT test to diagnose infection, genetic variation for susceptibility to *M. bovis* infection exists among dairy cows. Given the high PPV of the SICCT test, it is reasonable to conclude that both NVLs and VLs are infected with *M. bovis*, and therefore using SICCT test data to produce EBVs or gEBVs would ensure breeding for reduced susceptibility to *M. bovis* infection in cattle.

## Conclusions

This study identified genomic regions and chromosomes significantly associated with bTB resistance to infection in sampled Northern Ireland dairy cattle. RH mapping identified three loci distinct for the NVL phenotype on BTA17, BTA22 and BTA23, whilst a region on BTA13 was associated with the VL phenotype. The region with the strongest evidence of association on BTA23 contained immune-related genes, including *GCLC* and members of the *BoLA class IIb* region. Furthermore, the two QTLs on BTA22 and BTA23 were nearby bTB resistance QTLs identified in two other bTB GWAS studies. Chromosome heritability analysis attributed variance to six and thirteen chromosomes for the VL and NVL phenotypes, respectively and grouping the *M. bovis* outcomes (controls vs all cases) resulted in variance attributed to nine chromosomes. Furthermore, nine chromosomes were found to harbour variants that distinguished the NVLs and VLs. These findings suggest the host genetics of *M. bovis* infection outcomes is governed by distinctive genetic variation, along with shared genetic variants. It follows that there are at least two distinct outcomes to diagnosed *M. bovis* infection in dairy cattle, which may be indicative of different host responses or pathogen handling.

## Material and methods

### Sample collection

As described in Bermingham et al. [6], samples were taken from cattle at slaughter that tested positive under either the standard or severe interpretation of the SICCT test between August 2008 and June 2009 alongside Northern Ireland’s routine screening of herds. Descriptions of the SICCT test interpretations can be found in Lesslie and Herbert [44] and Morrison et al. [45], but to note the severe interpretation is adopted under certain conditions whereby the cut-off point is lowered to enhance the sensitivity of the test. As per routine bTB surveillance, SICCT-positive carcases were examined in the abattoir for signs of pathology and upon detection tissue was sampled for bacterial culturing and molecular typing of *M. bovis*. High-prevalence herds with sampled SICCT-positive cows were also sourced for SICCT-negative animals.

### Phenotype definition

Three phenotypes were defined based on the outcomes of the diagnostic tests SICCT and carcase abattoir examination. First, SICCT-positives, under either the standard or severe interpretation, were defined as cases. Next, cases were sub-categorised into (i) animals confirmed for *M. bovis* infection with evidence of pathology in the form of detectable lesions from carcase abattoir examination, positive culture and molecular confirmation of *M. bovis* (VLs) and (ii) animals unconfirmed for *M. bovis* infection as no lesions were detected from carcase abattoir examination (NVLs). Controls were SICCT-negatives on multiple occasions before, during and after a herd breakdown, and were sourced from high bTB prevalence herds (defined as herds that had a minimum of 2 VLs). In addition, longitudinal data were available and examined to ensure the healthy status of the controls over time [6].

### Selecting phenotype samples

VLs and controls samples were selected in a previous case–control study (see [6]). Since VLs comprised of both standard and severe reactors, NVLs were selected under both interpretations of the SICCT test. To have a set of epidemiologically comparable animals for the 2 case phenotypes, female NVLs were selected from herds already sampled for VLs [6]. A total of 873 NVLs met the above criteria, of which 625 and 248 were positive under the standard and severe interpretation of the SICCT test, respectively. Due to the lower cut-off threshold applied under the severe interpretation, the probability that a cow was truly infected when it tested positive under the severe interpretation was estimated as the PPV [46] for each herd: $PPV=\frac{Se. p}{\left( Se. p\right)+\left(1- Sp\right)\left(1- p\right)},$ where *Se* was the sensitivity, *Sp* was the specificity and *p* was the true prevalence: $p=\frac{p^{\prime }+ Sp-1}{Se+ Sp-1}$, where *p’* was the proportion of SICCT-positive animals of the total herd. Since some herds had SICCT test positive animals from more than test time-point between August 2008 and June 2009 the highest calculated herd prevalence from that timeframe was used. Values of 0.659 and 0.998 for the *Se* and *Sp* for the severe interpretation of the SICCT test were used, respectively, and fall within the range of estimates found in the literature [33]. A total of 223 severe reactors had a PPV ≥ 0.90 and were shortlisted for genotyping along with the 625 standard reactors.

### SNP chip genotyping and quality control

DNA from blood samples had been previously extracted (see [6]). After taking into account low DNA concentrations, 837 NVLs, comprising 616 standard and 221 severe reactors remained. From the previous bTB case–control study a total of 27 VLs that failed quality control due to a low call rate (<90%) were also re-genotyped [6]. In total, 864 samples were genotyped with the BovineHD Genotyping BeadChip (Illumina Inc., San Diego, CA, USA) by Edinburgh Genomics. A high-density SNP dataset was available (592 VLs and 559 controls [6]) and combined the two genotype datasets together comprised of 2015 animals genotyped at 777,962 SNP markers, categorised into 619 VLs, 837 NVLs and 559 controls.

Quality control of the data was implemented prior to analysis. Samples with greater than 10% missing genotypes and were removed. Pairs of animals with an average identity by state ≥0.90 were removed, carried out in GenABEL [47]. Marker reproducibility was assessed [48] by comparing the VLs re-genotyped in this study to their genotypes from the previous study [6]. Twenty re-genotyped VLs were used to contrast the SNP calls between the two genotype datasets. In total, 19,808 SNPs did not match between both the genotype datasets in one or more animal and these SNPs were removed (2.5% of SNPs). Next, the two genotype datasets were cleaned separately prior to merging [49]. SNPs were discarded if they were monomorphic (MAF < 0.05), had greater than 10% missing genotypes, deviated from Hardy-Weinberg Equilibrium at a critical rejection region of 8.1 × 10^−6^ and were on sex chromosomes. Following quality control the two genotype datasets were merged based on common SNPs. The final dataset comprised 1966 animals genotyped at 538,231 autosomal SNP markers, categorised into 607 VLs, 800 NVLs and 559 controls.

### Analysis

Genetic analyses were carried out where the bTB status (phenotypes) was coded as a binary trait (0, 1) for VLs (controls vs VLs), NVLs (controls vs NVLs), all cases (controls vs SICCT positives) and NVLs vs VLs.

Multi-dimensional scaling (MDS) was performed for each binary trait using the genomic relationship matrix (GRM) to assess population structure amongst animals using GenABEL [47]. No genetic structuring was observed amongst the animals or clustering of distinctive of the VLs, NVLs, standard SICCT reactors, severe SICCT reactors and controls (Additional file 6). Therefore, population stratification was not considered, other than to account for relatedness using the GRM.

Regional heritability (RH) mapping was conducted [20] to identify regions of genetic variance associated with *M. bovis* infection outcomes, carried out using DISSECT [50]. RH mapping of the whole genome was conducted using 100-SNP window sizes shifted every 50 SNPs and 50-SNP window sizes shifted every 25 SNPs. Fine RH mapping of chromosomes with QTLs detected above and just below significance levels was conducted using 30-SNP window sizes shifted every 15 SNPs. The GRM for each region was fitted separately as a random effect in a linear mixed model along with the whole-genome GRM:

$$ \mathbf{y}=\mathbf{W}\boldsymbol{\upalpha } +\mathbf{u}+{\mathbf{g}}_{\mathbf{r}}+\mathbf{e} $$

where **y** is a vector of phenotypes, **α** is a vector of fixed effects with its incidence matrix **W**, **u** is a vector of random additive genetic effects (the polygenic effect), **g** is a vector of the random regional additive genetic effects of SNPs in a pre-defined window and **e** is a vector of the random residual effects. Fixed effects included were breed (Holstein vs Friesian), age at the start of the herd breakdown-initiating SICCT test, year, season and test reason for the herd breakdown-initiating SICCT test and highest estimated herd bTB prevalence during the breakdown. Random effects were distributed as **u** ~ *N*(0, **G**σ^2^_a_), **g**_***r***_ ~ *N*(0, **Q**σ^2^_r_) and **e** ~ *N*(0, **I**σ^2^_e_) where matrices **G**, **Q** and **I** were the whole-genome GRM, a regional GRM using SNPs within a window and an identity matrix, respectively. Phenotypic variance was calculated as σ^2^_p_ = σ^2^_a_ + σ^2^_r_ + σ^2^_e_, where σ^2^_a_, σ^2^_e_ and σ^2^_r_ are the additive genetic, residual and regional additive genetic variance, respectively, and heritability for each region was estimated as h^2^_r_ = σ^2^_r_/σ^2^_p_. The null hypothesis was a linear mixed model without the regional additive genetic effect (i.e. only the polygenic effect was fitted):

$$ \mathbf{y}=\mathbf{W}\boldsymbol{\upalpha } +\mathbf{u}+\mathbf{e} $$

Significance at each window was assessed using the likelihood ratio test (LRT) calculated for the model fitting regional variance (fitting both whole-genome and regional GRM) against the null hypothesis of no regional variance (fitting only the whole-genome GRM). The test statistic was assumed to follow a chi-square distribution. Windows at the ends of chromosomes that did not meet the pre-defined window size were discarded. Bonferroni correction was implemented to account for multiple testing with half the number of windows used due to overlapping windows. After Bonferroni correction, LRT thresholds were 18.32 and 13.95 for the 100-SNP window scan, 19.51 and 15.26 for the 50-SNP window scan and 20.62 and 16.23 for the 30-SNP window scan, for genome-wide (*P* < 0.05) and suggestive significance (one false positive per genome scan), respectively.

To determine how genetic variation associated with *M. bovis* infection outcomes was apportioned across the bovine genome the variance attributable to each chromosome was estimated [51], implemented in GCTA [52]. A GRM for each chromosome was produced using its respective SNPs and chromosome heritability was calculated using a single chromosome decomposition approach. The GRM for each chromosome was fitted separately, along with the GRM for the whole genome excluding the target chromosome:

$$ \mathbf{y}=\mathbf{W}\boldsymbol{\upalpha } +{\mathbf{u}}_{-\mathbf{c}}+{\mathbf{g}}_{\mathbf{c}}+\mathbf{e} $$

where **g**_**c**_ is a vector of random genetic effects for chromosome *c*, with **g**_**c**_ ~ *N*(0, **G**_c_σ^2^_c_) where **G**_c_ is the GRM for chromosome *c* and **u**_**-c**_ is the whole-genome polygenic effect for the remaining SNPs distributed as *N*(0, **G**_-c_σ^2^_a(−c)_) where **G**_-c_ is the GRM for all SNPs excluding those on chromosome *c*. For chromosome *c* the phenotypic variance was calculated as σ^2^_p_ = σ^2^_a(−c)_ + σ^2^_c_ + σ^2^_e_ and heritability was estimated as h^2^_c_ = σ^2^_c_/σ^2^_p_.

If a trait is of a highly polygenic nature a strong linear relationship between chromosome length and the proportion of variance explained the chromosome, such as height, is expected [51]. A linear regression between the proportion of variance explained by each chromosome and chromosome length was carried out. Chromosome length in base pairs was taken from http://www.ncbi.nlm.nih.gov/genome?term=bos%20taurus, which used the UMD3.1.1 genome assembly.

A genome-wide association analysis was also carried and details of the methods employed is provided in the supplementary material (Additional file 1).

## Additional files

Additional file 1: Description of methods and results obtained from a genome-wide association study. (DOCX 16 kb)

Additional file 2: Significant SNPs using a linear mixed model for each case–control classifications for *M. bovis* infection. For each trait, the table shows chromosome, significant SNPs, reference SNP id number (rs id), position (in base pairs), the minor allele, minor allele frequency beta coefficient (substitution effect of the minor allele) and *P*-value of the GWAS analysis (DOCX 15 kb).

Additional file 3: Genome-wide association analysis for controls vs NVLs **a** Manhattan plot displaying the –log_10_(*P-* value) of association of each SNP with the phenotype with respect to genomic position and **b** Q-Q plot of observed *P*-values against the expected *P*-values with a genomic inflation factor of λ = 1.004. Genome-wide (*P* < 0.05) and suggestive significance (one false positive per genome scan) *P*-value thresholds are shown as a dashed and dotted line, respectively (PDF 2422 kb).

Additional file 4: Genome-wide association analysis for controls vs cases **a** Manhattan plot displaying the –log_10_(*P-* value) of association of each SNP with the phenotype with respect to genomic position and **b** Q-Q plot of observed *P*-values against the expected *P*-values with a genomic inflation factor of λ = 1.01. Genome-wide (*P* < 0.05) and suggestive significance (one false positive per genome scan) *P*-value thresholds are shown as a dashed and dotted line, respectively (PDF 2501 kb).

Additional file 5: Genome-wide association analysis for NVLs vs VLs **a** Manhattan plot displaying the –log_10_(*P-* value) of association of each SNP with the phenotype with respect to genomic position and **b** Q-Q plot of observed *P*-values against the expected *P*-values with a genomic inflation factor of λ = 1.005. Genome-wide (*P* < 0.05) and suggestive significance (one false positive per genome scan) *P*-value thresholds are shown as a dashed and dotted line, respectively (PDF 2438 kb).

Additional file 6: Multi-dimensional scaling (MDS) analysis using a similarity distance matrix calculated from the identity-by-descent genomic kinship matrix. Analysis was done for the each case/control classification with colours reflecting the different phenotypes and are plotted as **a** controls vs VLs, **b** controls vs NVLs, **c** controls vs cases and **d** NVLs vs VLs (PDF 43 kb).
