# Supplementary material for: Fine-mapping host genetic variation underlying outcomes to Mycobacterium bovis infection in dairy cows
Source: BMC Genomics. 2017 Jun 24;18:477. doi: 10.1186/s12864-017-3836-x (PMC5483290; doi:10.1186/s12864-017-3836-x)
Supplement: Supplementary file 1 — Description of methods and results obtained from a genome-wide association study. (DOCX 16 kb) [file 12864_2017_3836_MOESM1_ESM.docx]

**Description of methods and results used in the genome-wide association study**

*Methods*

Single-SNP association analyses were conducted using GEMMA [[1](#_ENREF_1), [2](#_ENREF_2)], which implements an exact mixed model approach to test for an association with a SNP effect whilst accounting for relatedness using the GRM in one step:

**y = Wα + xβ + u + e**

where **y** is a vector of phenotypic values (binary bTB status; 0: control, 1: case), **α** is a vector of fixed effects with its incidence matrix **W**, **x** is a vector of marker genotypes and **β** is the regression of the phenotype on the marker genotypes (the SNP effects), **u** is a vector of additive genetic effects (the polygenic effect) from the GRM and **e** is a vector of residual effects. . Distributions of the random effects were assumed to follow **u** ~ N(0, **G**σ^2^_a_) and **e** ~ N(0, **I**σ^2^_e_) where matrices **G** and **I** were the whole whole-genome GRM and an identity matrix, respectively. Phenotypic variance was calculated as σ^2^_p_ = σ^2^_a_ + σ ^2^_e_, where σ^2^*_a_* and σ^2^*_r_* are the additive genetic and residual variance, respectively, and it follows that heritability was estimated as h^2^ = σ^2^_a_ / σ^2^_p_. Fixed effects included were breed (Holstein vs Friesian, age at the start of the herd episode-initiating SICCT test, year, season and test reason for the breakdown-initiating SICCT test and highest estimated herd bTB prevalence during the breakdown. Significance of each SNP effect (regression coefficient) was determined using a Wald test. After Bonferroni correction, genome-wide and suggestive (one false positive per genome scan) significance thresholds were *P* < 9.3 x 10^-8^ and *P* < 1.85 x 10^-6^ which correspond to –log_10_(*P*) of 7.03 and 5.73, respectively. To discount spurious SNP associations due to systematic biases (experimental or other confounding factors) and/or population stratification, first, quantile-quantile (QQ) plots of observed p-values versus expected under the null hypothesis were plotted and, second, the genomic inflation factor (λ), the ratio of the median of the empirical test-statistic to the expected median, was calculated.

*Results*

Results from GWAS are presented in the supplementary material with a brief summary given here. SNPs at suggestive and genome-wide significance are listed in Additional file 2. For the four phenotype classifications, the Q-Q plot of observed against expected *P*-values for each SNP (Additional file 3b, 4b and 5b) did not reveal deviations from the null hypothesis, apart from SNPs at the tail end of the distribution, indicating that population structure was accounted. Genomic heritability estimates were 0.45 (± 0.06), 0.22 (± 0.04) and 0.29 (± 0.06) for the controls vs NVLs, controls vs cases and NVLs vs VLs, respectively. GWAS revealed 16 SNPs were significant at the genome-wide and suggestive significance levels on 12 chromosomes for the controls vs NVLs (Additional file 3a). For the controls-cases, 1 SNP on BTA7 was at suggestive significance (Additional file 4a). When considering the NVLs-VLs, 10 SNPs were significant at the genome-wide and suggestive significance levels (Additional file 5a). Significant SNPs for one of the phenotype classification were not replicated for another given phenotype classification. In addition, the significant SNPs did not reveal any clear regions of associations as they were not neighbouring or in close proximity to one another.

1. Zhou X, Stephens M: **Genome-wide efficient mixed-model analysis for association studies**. *Nature Genetics* 2012, **44**(7):821-U136.

2. Zhou X, Stephens M: **Efficient multivariate linear mixed model algorithms for genome-wide association studies**. *Nature Methods* 2014, **11**(4):407.
