# Supplementary material for: Fine-mapping host genetic variation underlying outcomes to Mycobacterium bovis infection in dairy cows
Source: BMC Genomics. 2017 Jun 24;18:477. doi: 10.1186/s12864-017-3836-x (PMC5483290; doi:10.1186/s12864-017-3836-x)
Supplement: Supplementary file 2 — Significant SNPs using a linear mixed model for each case–control classifications for M. bovis infection. For each trait, the table shows chromosome, significant SNPs, reference SNP id number (rs id), position (in base pairs), the minor allele, minor allele frequency beta coefficient (substitution effect of the minor allele) and P-value of the GWAS analysis (DOCX 15 kb). [file 12864_2017_3836_MOESM2_ESM.docx]

Additional file 2 Summary of significant SNPs using a linear mixed model implemented in GEMMA. For each trait, the table shows chromosome, significant SNPs, reference SNP id number (rs id), position (in base pairs), the minor allele, minor allele frequency beta coefficient (substitution effect of the minor allele) and P-value of the GWAS analysis.

| **Trait** | **Chr** | **SNP** | **rs id** | **Pos (bp)** | **Minor allele** | **MAF** | **Beta coeff.** | **p-value** |
| --- | --- | --- | --- | --- | --- | --- | --- | --- |
| **Con-NVLs** |  |  |  |  |  |  |  |  |
|  | 1 | BovineHD0100003731 | rs135210818 | 11 952 874 | G | 0.115 | -0.16 ± 0.03 | 1.788822e-08 |
|  | 5 | BovineHD0500028820 | rs109319387 | 100 617 453 | A | 0.286 | 0.12 ± 0.02 | 3.367736e-07 |
|  | 6 | BovineHD0600005947 | rs137043916 | 21 540 903 | G | 0.071 | 0.18 0.04 | 1.162041e-06 |
|  | 10 | BovineHD1000015507 | rs109596264 | 51 815 028 | A | 0.112 | 0.17 ± 0.03 | 4.726514e-08 |
|  | 13 | BovineHD1300022538 | rs135506155 | 77 857 224 | A | 0.178 | 0.13 ± 0.03 | 8.953350e-07 |
|  | 13 | BovineHD1300024032 | rs43750684 | 82 783 349 | A | 0.187 | -0.12 ± 0.02 | 1.067213e-06 |
|  | 14 | BovineHD1400002809 | rs134776510 | 9 961 106 | G | 0.114 | -0.16 ± 0.03 | 1.259935e-07 |
|  | 16 | BovineHD1600007709 | rs134979205 | 27 600 567 | A | 0.092 | 0.16 ± 0.03 | 1.649913e-06 |
|  | 20 | BovineHD2000005438 | rs41937084 | 18 085 734 | A | 0.099 | 0.17 ± 0.04 | 1.204415e-06 |
|  | 20 | BovineHD2000007406 | rs110688408 | 24 610 181 | A | 0.077 | 0.17 ± 0.04 | 1.341697e-06 |
|  | 20 | BovineHD2000012746 | rs42738279 | 44 875 858 | G | 0.097 | -0.16 ± 0.03 | 1.532765e-06 |
|  | 23 | BovineHD2300001697 | rs134180999 | 6 774 223 | G | 0.499 | 0.09 ± 0.02 | 9.975132e-07 |
|  | 23 | BovineHD2300001699 | rs133043914 | 6 776 117 | G | 0.499 | 0.09 ± 0.02 | 9.975132e-07 |
|  | 26 | BovineHD2600007166 | rs132780515 | 26 951 677 | A | 0.116 | 0.15 ± 0.03 | 9.714602e-07 |
|  | 27 | BovineHD2700006925 | rs137092061 | 24 632 452 | A | 0.116 | 0.15 ± 0.03 | 4.370136e-07 |
|  | 29 | BovineHD2900001933 | rs42160303 | 6 921 472 | A | 0.418 | 0.09 ± 0.02 | 1.723493e-06 |
|  |  |  |  |  |  |  |  |  |
| **Con-Cases** |  |  |  |  |  |  |  |  |
|  | 7 | BovineHD0700027258 | rs132819531 | 93 337 531 | G | 0.163 | -0.10 ± 0.02 | 1.550511e-06 |
|  |  |  |  |  |  |  |  |  |
| **Nvls-VLs** |  |  |  |  |  |  |  |  |
|  | 4 | BovineHD0400007904 | rs109425434 | 27 315 392 | A | 0.128 | -0.14 ± 0.03 | 1.261540e-06 |
|  | 4 | BovineHD0400024973 | rs137305245 | 90 164 413 | G | 0.116 | -0.15 ± 0.03 | 3.042958e-07 |
|  | 5 | BovineHD0500002383 | rs110035490 | 8 439 953 | G | 0.093 | 0.16 ± 0.03 | 1.453009e-06 |
|  | 5 | BovineHD0500031607 | rs110995121 | 109 684 790 | A | 0.126 | -0.14 ± 0.03 | 1.566874e-06 |
|  | 7 | BovineHD0700009365 | rs133743609 | 32 717 852 | G | 0.170 | 0.13 ± 0.03 | 5.236233e-07 |
|  | 12 | BovineHD1200024894 | rs109073153 | 85 877 146 | A | 0.286 | -0.11 ± 0.02 | 9.701574e-07 |
|  | 21 | BovineHD2100002827 | rs110788696 | 11 392 195 | G | 0.090 | 0.17 ± 0.03 | 2.278514e-07 |
|  | 22 | BovineHD4100015466 | rs29014724 | 10 522 003 | A | 0.377 | -0.11 ± 0.02 | 9.174838e-07 |
|  | 29 | BovineHD2900012372 | rs42185418 | 40 888 815 | A | 0.490 | -0.10 ± 0.02 | 7.227243e-07 |
|  | 29 | BovineHD2900012864 | rs132936077 | 42 687 176 | A | 0.076 | -0.19 ± 0.04 | 2.883408e-07 |
