# Supplementary figures and images for: Fine-mapping host genetic variation underlying outcomes to Mycobacterium bovis infection in dairy cows
Source: BMC Genomics. 2017 Jun 24;18:477. doi: 10.1186/s12864-017-3836-x (PMC5483290; doi:10.1186/s12864-017-3836-x)

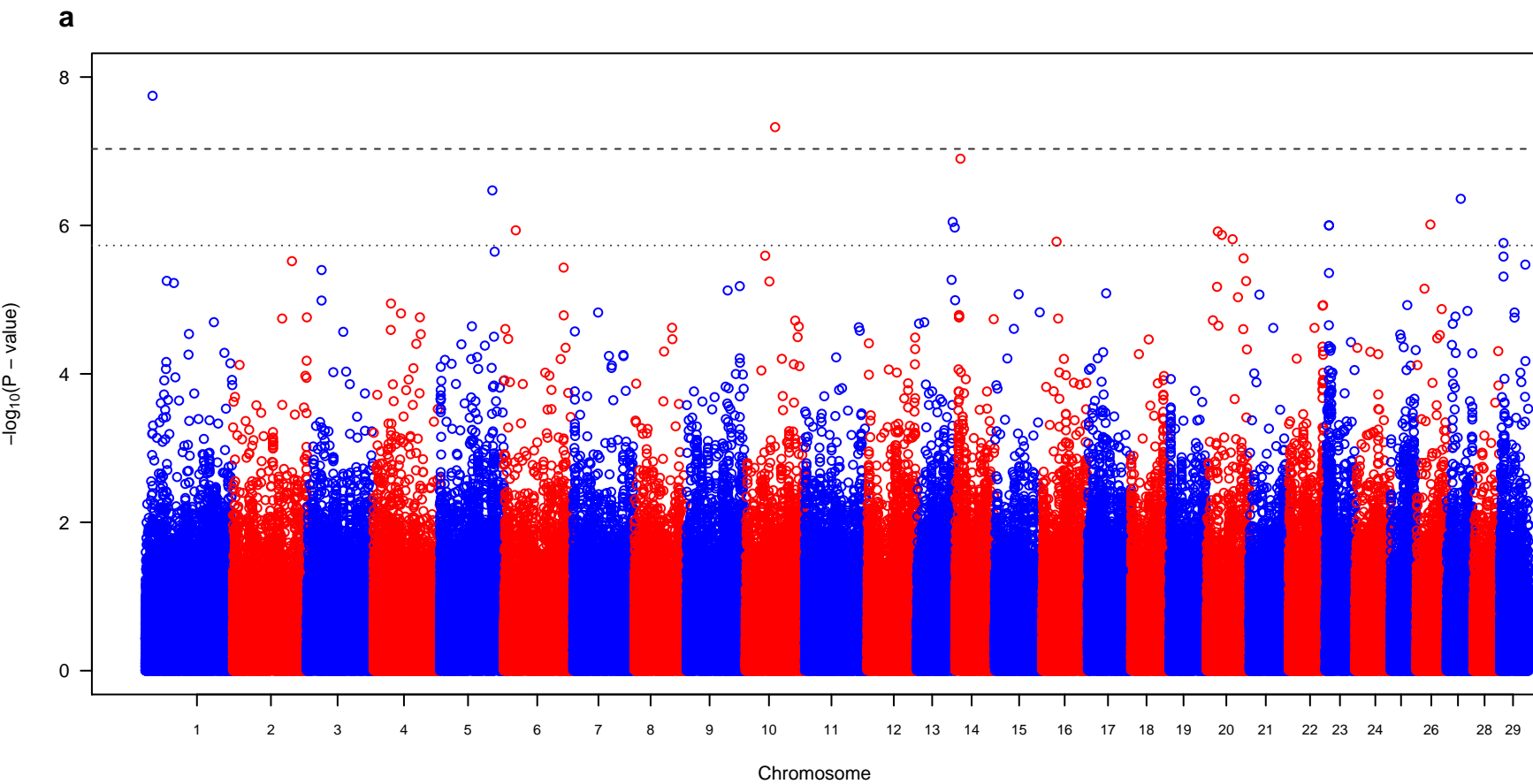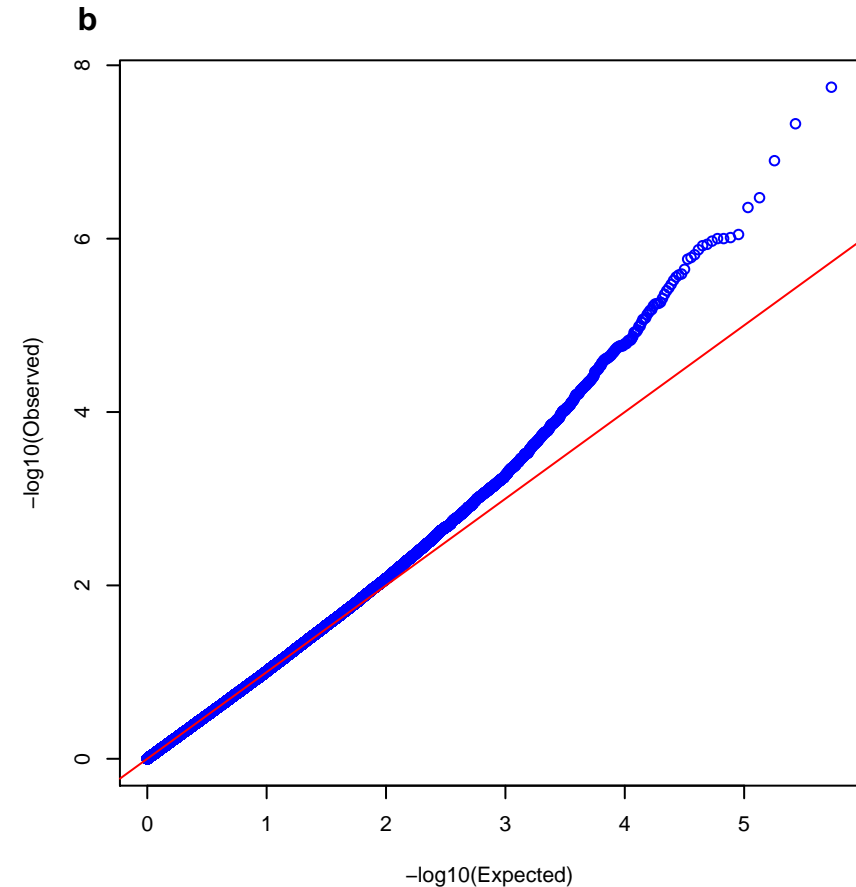

Supplement: Supplementary file 3 — Genome-wide association analysis for controls vs NVLs a Manhattan plot displaying the –log10(P- value) of association of each SNP with the phenotype with respect to genomic position and b Q-Q plot of observed P-values against the expected P-values with a genomic inflation factor of λ = 1.004. Genome-wide (P < 0.05) and suggestive significance (one false positive per genome scan) P-value thresholds are shown as a dashed and dotted line, respectively (PDF 2422 kb). [file 12864_2017_3836_MOESM3_ESM.pdf]

**a**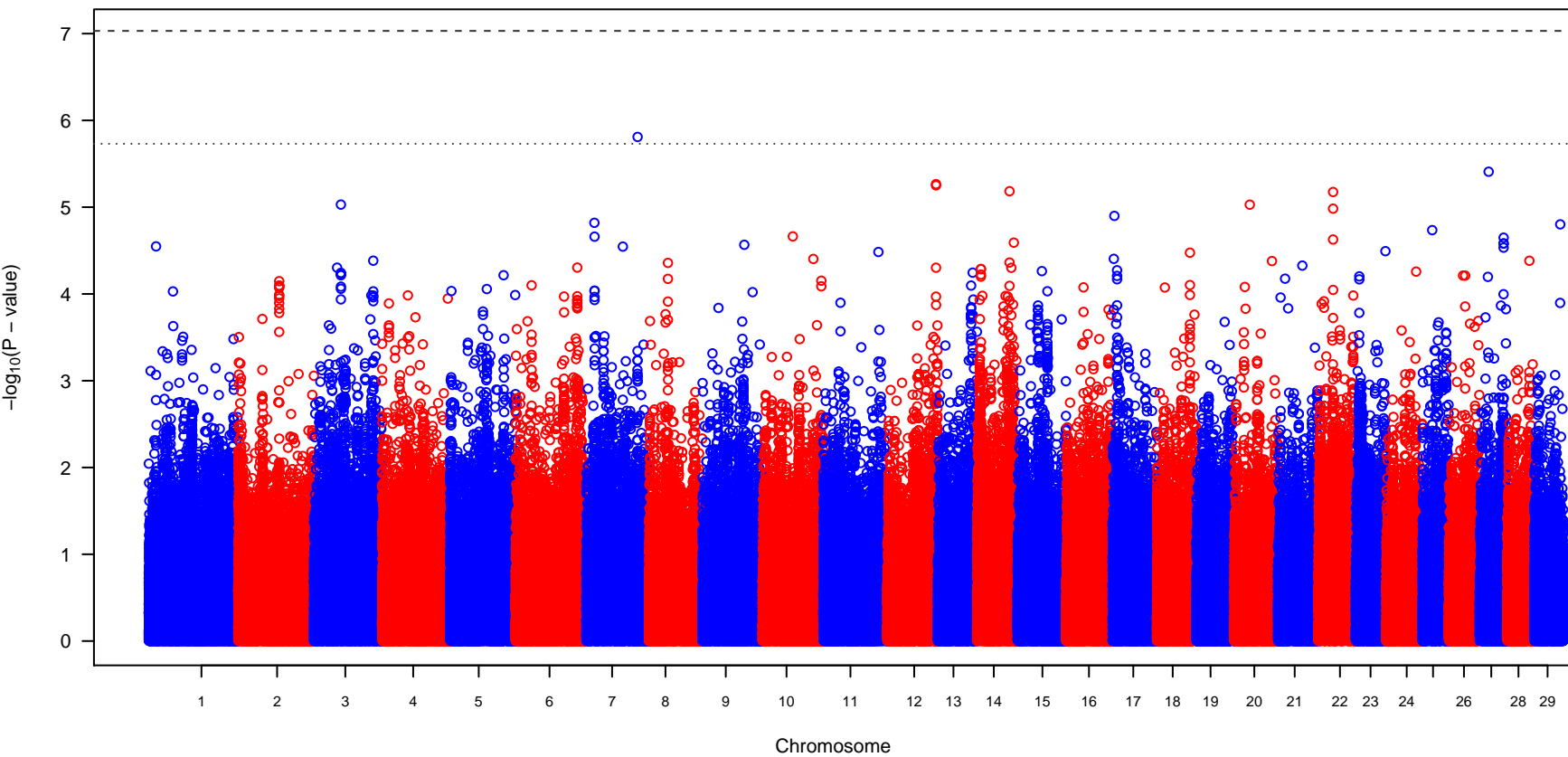**b**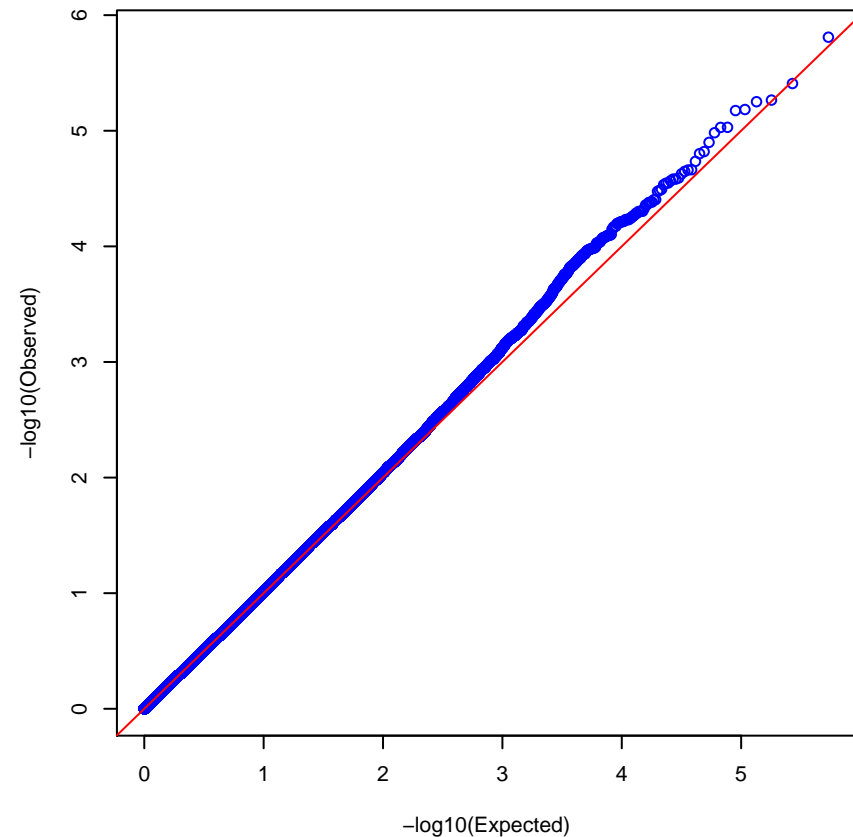

Supplement: Supplementary file 4 — Genome-wide association analysis for controls vs cases a Manhattan plot displaying the –log10(P- value) of association of each SNP with the phenotype with respect to genomic position and b Q-Q plot of observed P-values against the expected P-values with a genomic inflation factor of λ = 1.01. Genome-wide (P < 0.05) and suggestive significance (one false positive per genome scan) P-value thresholds are shown as a dashed and dotted line, respectively (PDF 2501 kb). [file 12864_2017_3836_MOESM4_ESM.pdf]

**a**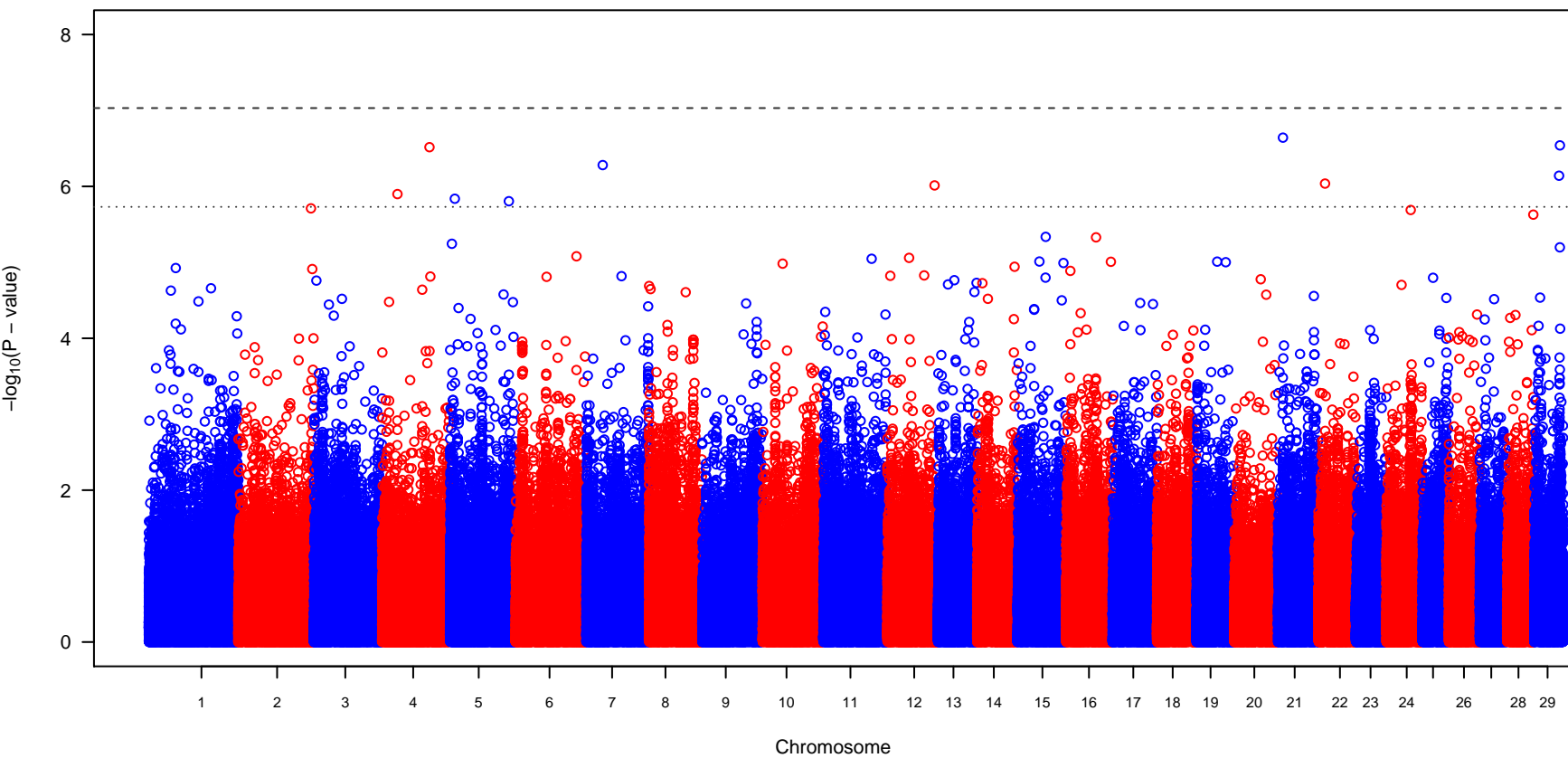**b**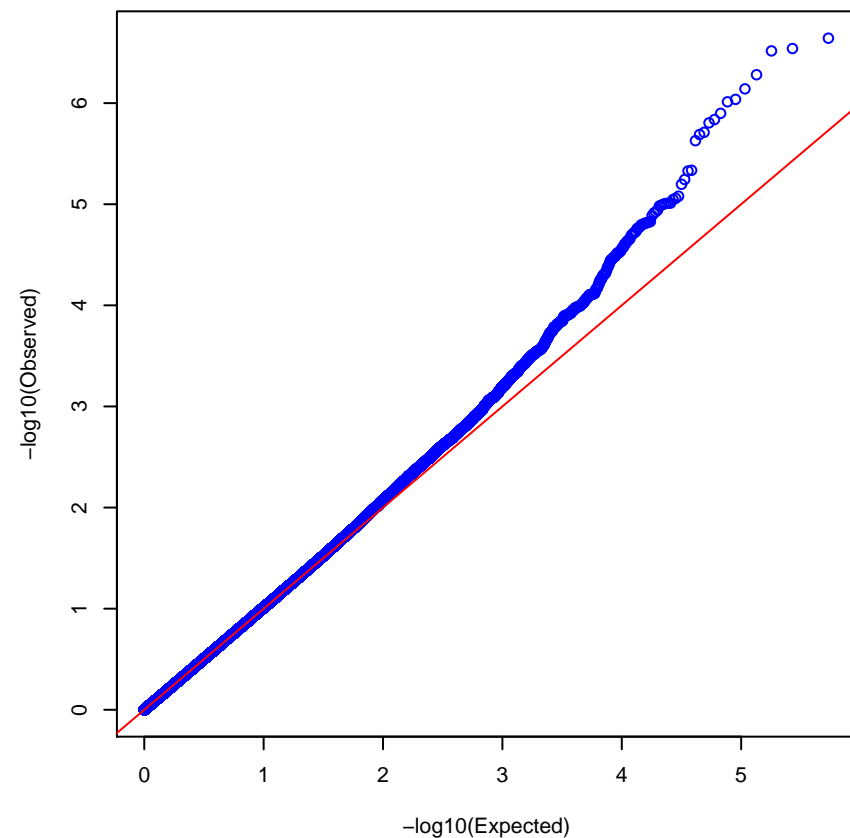

Supplement: Supplementary file 5 — Genome-wide association analysis for NVLs vs VLs a Manhattan plot displaying the –log10(P- value) of association of each SNP with the phenotype with respect to genomic position and b Q-Q plot of observed P-values against the expected P-values with a genomic inflation factor of λ = 1.005. Genome-wide (P < 0.05) and suggestive significance (one false positive per genome scan) P-value thresholds are shown as a dashed and dotted line, respectively (PDF 2438 kb). [file 12864_2017_3836_MOESM5_ESM.pdf]

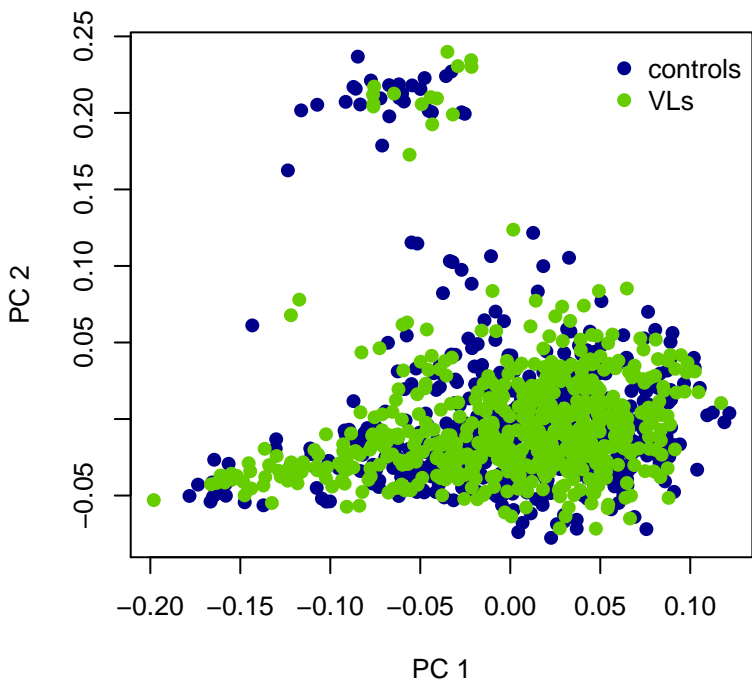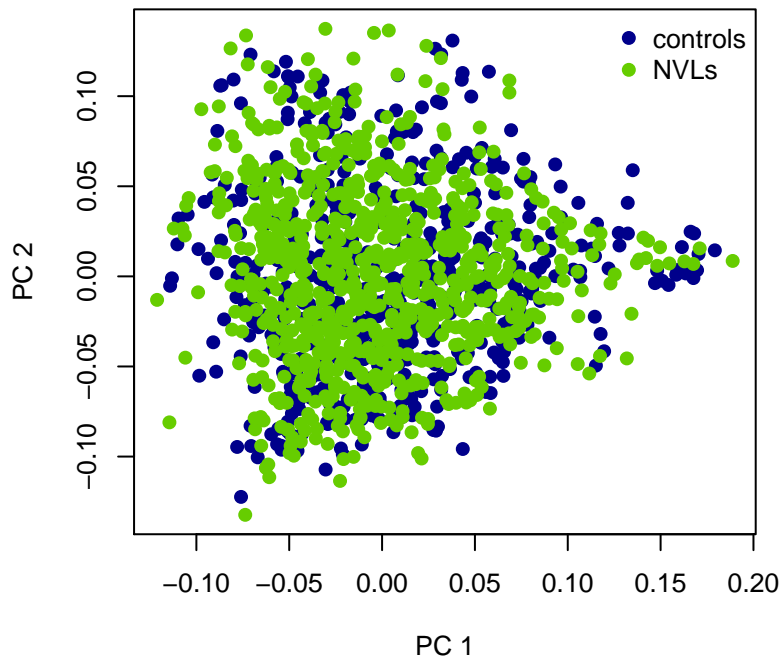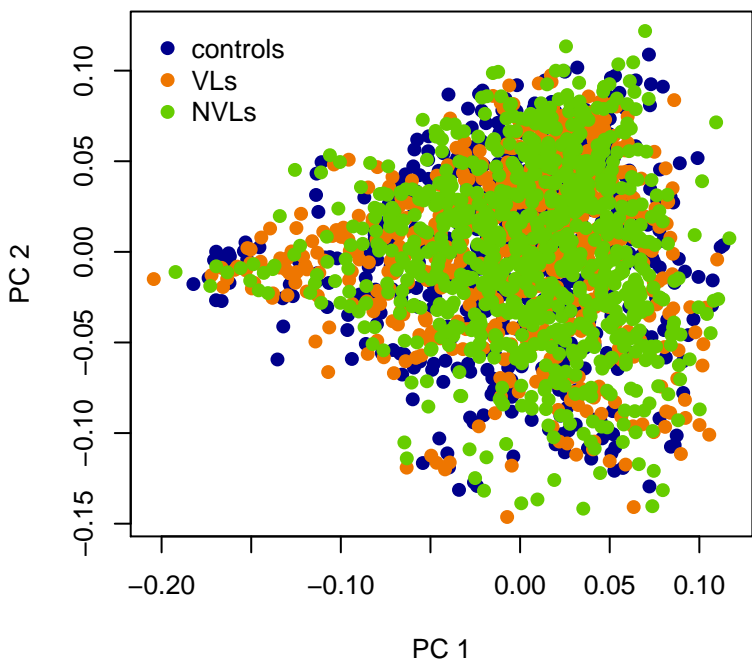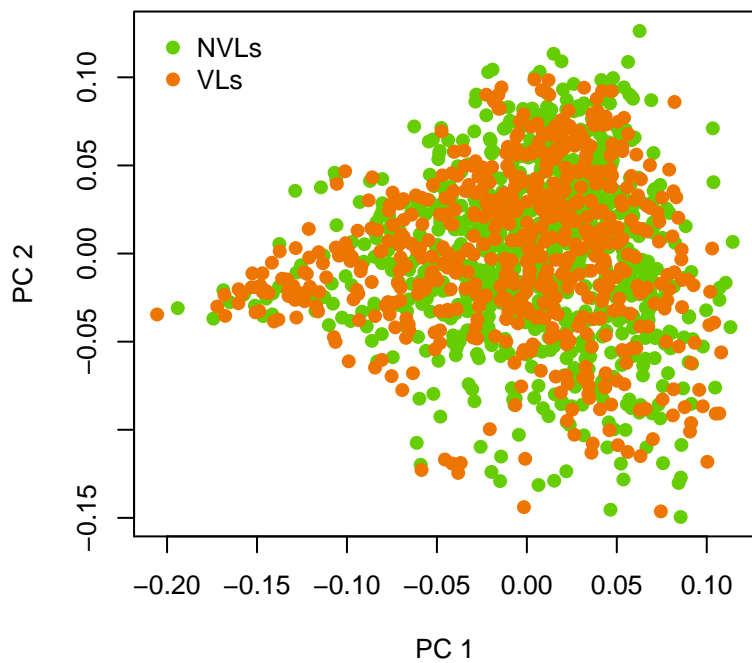

Supplement: Supplementary file 6 — Multi-dimensional scaling (MDS) analysis using a similarity distance matrix calculated from the identity-by-descent genomic kinship matrix. Analysis was done for the each case/control classification with colours reflecting the different phenotypes and are plotted as a controls vs VLs, b controls vs NVLs, c controls vs cases and d NVLs vs VLs (PDF 43 kb). [file 12864_2017_3836_MOESM6_ESM.pdf]
